# Renal amyloidosis: a new time for a complete diagnosis

**DOI:** 10.1590/1414-431X2022e12284

**Published:** 2022-10-03

**Authors:** V.A. Feitosa, P.D.M.M. Neves, L.B. Jorge, I.L. Noronha, L.F. Onuchic

**Affiliations:** 1 Divisão de Nefrologia, Faculdade de Medicina, Universidade de São Paulo, São Paulo, SP, Brasil; 2 Divisão de Medicina Molecular, Faculdade de Medicina, Universidade de São Paulo, São Paulo, SP, Brasil

**Keywords:** Renal amyloidosis, Amyloid typing, Proteomics, Mass spectrometry, Hereditary amyloidosis

## Abstract

Amyloidoses are a group of disorders in which soluble proteins aggregate and deposit extracellularly in tissues as insoluble fibrils, causing organ dysfunction. Clinical management depends on the subtype of the protein deposited and the affected organs. Systemic amyloidosis may stem from anomalous proteins, such as immunoglobulin light chains or serum amyloid proteins in chronic inflammation or may arise from hereditary disorders. Hereditary amyloidosis consists of a group of rare conditions that do not respond to chemotherapy, hence the identification of the amyloid subtype is essential for diagnosis, prognosis, and treatment. The kidney is the organ most frequently involved in systemic amyloidosis. Renal amyloidosis is characterized by acellular pathologic Congo red-positive deposition of amyloid fibrils in glomeruli, vessels, and/or interstitium. This disease manifests with heavy proteinuria, nephrotic syndrome, and progression to end-stage kidney failure. In some situations, it is not possible to identify the amyloid subtype using immunodetection methods, so the diagnosis remains indeterminate. In cases where hereditary amyloidosis is suspected or cannot be excluded, genetic testing should be considered. Of note, laser microdissection/mass spectrometry is currently the gold standard for accurate diagnosis of amyloidosis, especially in inconclusive cases. This article reviews the clinical manifestations and the current diagnostic landscape of renal amyloidosis.

## Introduction

Amyloidosis is a disease caused by extracellular deposition of amyloid material, an insoluble fibrillary compound formed by misfolded proteins that acquire a self-aggregation capacity, ultimately leading to tissue damage (1). By acquiring the conformation of β-pleated sheets, such proteins give rise to hydrophobic, insoluble, proteolysis-resistant pathogenic fibrils. The diameter of these fibrils varies from 7 to 12 nm. These structures have a high affinity for the Congo red dye, which results in positive birefringence when viewed under polarized light. This finding is, in fact, pathognomonic for amyloidosis (2).

The formation of amyloid fibrils involves a combination of different factors, including a sustained increase in protein concentration, a triggering factor for protein misfolding, such as a genetic variant, and/or proteolytic remodeling of a protein into an amyloidogenic fragment (3). Notably, amyloid deposits are formed by the interaction of these fibrils with the amyloid P component, apolipoprotein E, and glycosaminoglycans, which are essential to the assembly and maintenance of amyloid deposits in tissues. Amyloid deposition may be localized or systemic, an incidental finding, or rapidly lethal. To date, approximately 36 amyloidogenic proteins have been identified in humans. Their corresponding clinical manifestations depend on the affected organ as well as on genetic and environmental factors (3).

Systemic amyloidoses are classified according to the amyloid precursor, following a nomenclature proposed by the International Society of Amyloidosis (Table 1) (4). The most prevalent forms of renal amyloidosis are light chain and amyloid A amyloidosis (AL and AA, respectively), although AL is far more prevalent than AA amyloidosis. In AL amyloidosis, an anomalous immunoglobulin (Ig) fragment (kappa or lambda light chain) is produced by abnormal clones of plasma cells (5). AA amyloidosis, on the other hand, is caused by the serum amyloid A (SAA) protein, which is produced by the liver in response to chronic inflammatory processes (6). In addition, leukocyte chemotactic factor 2 amyloidosis (ALECT2) is a recently described type of amyloidosis secondary to LECT2, a protein synthesized mainly by the liver and a chemotactic factor for neutrophils. Hereditary amyloidoses, in turn, are rarer forms of renal amyloidosis that result from variants in specific genes, causing the encoded protein product to acquire amyloidogenic properties. Such proteins include fibrinogen A α-chain (AFib), transthyretin (ATTR), apolipoproteins AI, AII, CII, and CIII, gelsolin (AGel), and lysozyme (ALys) (7,8).

**Table 1:** Amyloid fibril proteins and corresponding precursors in systemic amyloidosis.

	Precursor protein	Acquired or Hereditary	Target organs
AL	Immunoglobulin light chain	A, H	All organs, except CNS
AH	Immunoglobulin heavy chain	A	All organs, except CNS
AA	Serum amyloid A	A, H	Kidney, liver
ATTRwt	Transthyretin, wild type	A	Heart, lung, ligaments,
ATTRv	Transthyretin, variants	H	Tenosynovium PNS, ANS, heart, eye, leptomeninges
Aβ2M	β2-microglobulin, wild type	A	Musculoskeletal system
	β2-microglobulin, variants	H	Musculoskeletal system
AApoAI	Apolipoprotein AI, variants	H	Heart, liver, kidney, PNS, testis, larynx, skin
AApoAII	Apolipoprotein AII, variants	H	Kidney
AApoAIV	Apolipoprotein AIV, wild type	A	Kidney medulla and systemic
AApoCII	Apolipoprotein CII, variants	H	Kidney
AApoCIII	Apolipoprotein CIII, variants	H	Kidney
AGel	Gelsolin, variants	H	Kidney, PNS, cornea
ALys	Lysozyme, variants	H	Kidney
ALECT2	Leukocyte chemotactic factor 2	A	Kidney
AFib	Fibrinogen A α-chain, variants	H	Kidney (primarily)
ACys	Cystatin C, variants	H	CNS, PNS, skin
ABri	ABriPP, variants	H	CNS

CNS: central nervous system; PNS: peripheral nervous system; ANS: autonomic nervous system.

Early diagnosis is fundamental for therapeutic success in amyloidosis. In case of clinical suspicion and histological confirmation by Congo red staining showing apple green birefringence deposits under polarized light microscopy, it is mandatory to identify the precursor protein, usually by immunofluorescence (IF) and/or immunohistochemistry (IHC). Typing the amyloid component by laser microdissection/mass spectrometry, however, has become the gold standard methodology in the last decade, and constitutes an essential step in initially indeterminate cases (9).

## Systemic amyloidosis

**Figure 1:**
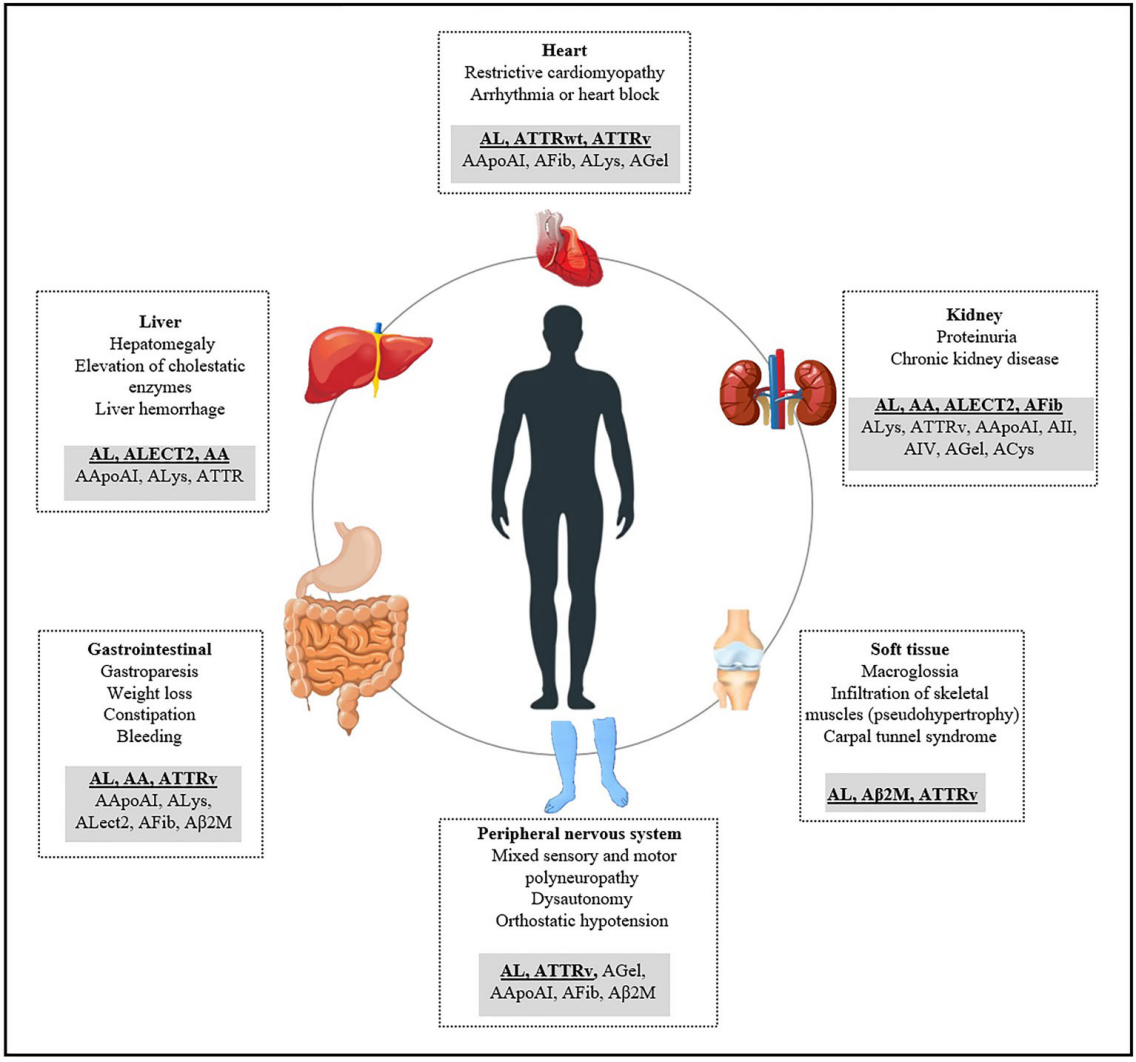
Subtypes of amyloidoses and affected organs/tissues. Bold type and underline indicate subtypes that most affect the organs.

Amyloidosis usually affects individuals from the fifth decade of life onwards, with rare exceptions. The deposits can occur in virtually any organ, and clinical manifestations are not specific to the amyloidosis subtypes, which may delay diagnosis and hinder treatment. The organs/systems most often affected are the heart, kidneys, peripheral nerves, and gastrointestinal tract. Preferential involvement of an organ, in turn, may suggest the pathogenic fibril type (Figure 1).

Cardiac involvement is observed in 50 to 80% of patients with AL amyloidosis and is a dominant finding in ATTR, often including restrictive cardiomyopathy and arrhythmias (5,10). This may be also observed in other hereditary amyloidoses, such as AApoAI, but is rare in AA amyloidosis. Peripheral neuropathy is found mainly in AL, ATTR, and AApoAI amyloidoses, most frequently affecting the axons (11). This is initially characterized by the appearance of paresthesia and loss of sensitivity to cold or heat. Autonomic neuropathy develops as the disease progresses, potentially leading to neurogenic bladder, early male impotence, postural hypotension, and gastroparesis. Liver involvement often presents as an increase in canalicular enzymes and hepatomegaly, while gastrointestinal tract disorders are usually associated with diarrhea, constipation, and intestinal bleeding (3). Although uncommon, soft tissues may also be affected, typically in AL amyloidosis, potentially causing macroglossia, periorbital purpura, muscular pseudohypertrophy, and salivary gland infiltration. Carpal tunnel syndrome is an early and common symptom in ATTR, affecting a significant number of these patients at an older age. A history of carpal tunnel syndrome associated with heart failure in older adult patients should, in fact, raise suspicion of ATTR wild-type amyloidosis (ATTRwt), previously known as senile systemic amyloidosis (1,7,11).

Serum amyloid P component (SAP) is a plasma protein present in all amyloid deposits. Based on this concept, scintilography with ^123^I-labeled SAP has been employed to evaluate organ involvement and the amyloid distribution profile in systemic amyloidosis. This method is successful in detecting amyloid in most AA and AL patients, but not in ATTR (12).

Despite the aforementioned heterogeneity of amyloidosis, and regardless of the affected organ, the diagnosis of amyloidosis requires the histological demonstration of amyloid deposits, usually by evaluation of the affected organs. Biopsy procedures in heart, kidney, and liver, however, may be associated with significant rates of complication, and are sometimes replaced or preceded by biopsy screening protocols of abdominal fat aspirate, salivary gland, and rectal mucosa. Although overall sensitivities vary from 70-90%, this approach may be especially useful to diagnose AL amyloidosis (3). Since Congo red staining is not a routine histological assessment, the diagnosis may be missed in the absence of clinical suspicion and warning signs (Table 2). To avoid missing such a diagnosis, systemic amyloidosis should be suspected in adults with nephrotic proteinuria, restrictive heart failure, neuropathy, and gastrointestinal symptoms/hepatomegaly with no apparent cause.

**Table 2:** Signs to investigate amyloidosis.

1. Nephrotic syndrome in adults (>40 years)
2. Restrictive cardiomyopathy
3. Peripheral neuropathy
4. Hepatomegaly
5. Monoclonal gammopathy of undetermined significance with:
a. Autonomic neuropathy
b. Unexplained fatigue
c. Edema
d. Unexplained weight loss

Adapted from Juneja and Pati (Ref. 67; doi: 10.1007/s12288-019-01208-4).

## Renal amyloidosis

The kidney is the primary or one of the main organs affected in AL, AA, ALECT2, and AFib amyloidosis (7). The most common clinical presentation is proteinuria, mainly composed of albumin, which can vary from subnephrotic to massive proteinuria. Isolated hematuria is uncommon (13,14). AL amyloidosis is the most frequent subtype of renal amyloidosis worldwide, however, epidemiological differences are observed among different countries. AL amyloidosis is more prevalent in developed countries, while AA amyloidosis prevails in developing countries due to the high prevalence of infectious diseases (15). In a Brazilian study including 110 cases of biopsy-proven systemic amyloidosis, 60 patients had renal involvement and immunoglobulin-related amyloidosis was predominant (16). Along this line, a Mayo Clinic series comprising 474 patients with histologically proven renal amyloidosis revealed Ig-related amyloidosis in the vast majority of the cases (86%), followed by 7% of AA, 3% of ALECT2, and 1% of AFib (17). It must be noted, however, that AFib is the leading cause of hereditary renal amyloidosis in Europe and the United States (18,19).

In adult cases of nephrotic syndrome, especially over 50 years of age, it is imperative to include renal amyloidosis in the differential diagnosis, as such cases are not infrequently misdiagnosed as minimal change disease when associated with mild or inadequately evaluated amyloid deposition. AL amyloidosis tends to present with a more pronounced nephrotic syndrome and hypotension compared to non-AL forms. Treating edema is often challenging, particularly in AL amyloidosis, as cardiac, vascular, and autonomic nervous system involvement leads to fluid retention and hypotension that is difficult to manage (7). Deposits confined to the tubulointerstitial or vascular compartments are observed in certain types of renal amyloidosis, resulting in progressive renal dysfunction with mild or absent proteinuria. This type of clinical presentation is observed in AApoAI, AApoAII, and ALECT2. Although rare, diabetes insipidus and Fanconi's syndrome have been described as a result of amyloid deposits in the collecting ducts and proximal convoluted tubules, respectively (14,20,21).

## Types of renal amyloidoses

### Immunoglobulin amyloidosis

Immunoglobulin amyloidosis results from anomalous immunoglobulin fragments with amyloidogenic properties, produced by monoclonal plasma cell disorders. Since the immunoglobulin structure comprises two light chains and two heavy chains, immunoglobulin-related amyloidoses are classified as light chain amyloidosis (AL), accounting for approximately 94% of cases; heavy and light chain amyloidosis (AHL), 5% of cases; and heavy chain amyloidosis (AH), 1% of cases (17).

According to different case series, AL clinically affects the kidneys in 50-80% of the patients (7,22). Other studies report proteinuria in approximately 75% of AL cases, nephrotic syndrome in ∼50%, and renal dysfunction in ∼20%, at the time of diagnosis (22). In AL amyloidosis, hypertension is uncommon, with a tendency toward hypotension and orthostasis. The diagnosis is often established based on a renal biopsy or following the development of proteinuria (>0.5 g/24 h) in a patient with a previous diagnosis of AL (10). A study including more than 400 renal biopsies from patients with AL amyloidosis identified amyloid deposits in glomeruli (97%), vessels (56%), interstitium (58%), and tubular basement membrane (8%), findings that explain why proteinuria constitutes an almost universal feature of this disease (17). Cardiac involvement is present in almost 80% of such patients. Peripheral and autonomic neuropathy are also common manifestations. Although ∼40% of AL patients display >10% of plasma cells in their bone marrow, only 10-20% of them meet criteria for multiple myeloma (MM) (23).

Investigation of AL requires the identification of monoclonal Ig. This detection is most often achieved using serum and urine protein electrophoresis, serum and urine protein immunofixation, and/or serum free light chain (sFLC) dosage, techniques that are sensitive to identification of a monoclonal Ig component in 65, 73, and 88% of cases, respectively (24). It must be emphasized that the joint use of such assays allows diagnosis of all patients with MM and 97% of AL cases (25). Due to the low amount of circulating monoclonal protein usually observed in AL amyloidosis compared to MM, immunofixation is often required to detect the paraprotein in AL patients. Quantification of sFLC using the Freelite assay, either with nephelometric or turbidimetric immune methods, is important not only to confirm the presence of light chains but also to monitor disease and assess response to treatment. In addition to deposits in glomeruli, interstitium, and/or vessels on light microscopy, renal biopsy immunofluorescence analysis of renal biopsy reveals light chain Ig (lambda in 70% of cases). Depositions of heavy chain Ig may be rarely identified, most often IgG. Electron microscopy also confirms the presence of amyloid fibrils (7,26,27). It is important to confirm the diagnosis of AL amyloidosis with identification of the corresponding monoclonal paraprotein, measurement of sFLC, and bone marrow biopsy.

Proteinuria >5 g/24 h and eGFR <50 mL/min at diagnosis have been associated with progression to dialysis in 60 and 85% of AL patients at 3 years, respectively (28–31). Of note, renal failure limits the therapeutic options. Whereas the hematologic response assessed by the rate of sFLC reduction is associated with renal and overall survival, patients that survive can still progress to end-stage kidney disease (ESKD). Therefore, early identification of renal response is essential, as it guides decisions on intensifying or modifying treatment (30).

Patients with systemic amyloid AL require therapy to prevent progressive organ failure. Stem cell transplant (SCT) is preferred, yet only 20–25% of patients are eligible. Patients not eligible for SCT can be offered melphalan-dexamethasone or cyclophosphamide-bortezomib-dexamethasone (CyBorD) (32). In a new approach, the addition of daratumumab (a human CD38-targeting antibody) to CyBorD was associated with higher frequencies of hematologic response and survival with no major organ dysfunction (33).

### Amyloid protein A amyloidosis

AA amyloidosis results from a persistently high production of serum amyloid A (SAA), an acute-phase reactant produced by hepatocytes in response to chronic inflammatory conditions. Inflammatory processes lead to systemic release of pro-inflammatory cytokines, such as tumor necrosis factor (TNF)-α, interleukin (IL)-1ß, and IL-6, which stimulate the production of SAA, activate the inflammasome cascade, and promote the recruitment of IL-17-producing T lymphocytes. Such processes include chronic infections, such as tuberculosis, osteomyelitis, schistosomiasis or bronchiectasis, and chronic inflammatory diseases, such as rheumatoid arthritis, vasculitis, Crohn's disease, or periodic febrile syndromes (e.g., familial Mediterranean fever, FFM) (6). For reasons not yet well understood, AA only occurs in a small proportion of patients with chronic inflammatory diseases. In Caucasians, polymorphisms in the *SAA1* and *SAA2* (serum amyloid A1 and A2) genes have been implicated in the pathogenesis of AA amyloidosis in individuals with FFM (34). Idiopathic forms represent a significant and increasing percentage (15-20%) of all diagnosed cases of AA amyloidosis. Recently, Sikora et al. identified a family suffering from primary AA amyloidosis due to a chr11: 18287683 T>C mutation in the *SAA1* promoter, with overexpression of amyloidogenic SAA1.1 protein leading to organ involvement, in the absence of inflammation (35).

The distribution of AA amyloidosis varies worldwide and, together with AL, constitute the main causes of renal amyloidosis in developing countries (15). A reduction in AA incidence has been observed in recent decades, reflecting the therapeutic advances and improvements in treating chronic inflammatory diseases (13,15). AA patients usually have inadequately controlled chronic inflammatory diseases, with high levels of C-reactive protein and SAA. These levels, in turn, can be useful to monitor the response to treatment. As the primarily affected organ, the kidneys are involved in 90% of cases, leading to massive proteinuria in approximately 95% of cases and to nephrotic syndrome in ∼75%. Up to 10% of patients have ESKD at the time of diagnosis, while progressive renal dysfunction will occur if the underlying disease remains poorly controlled (6,36). The liver is the second most affected organ. Unlike AL and ATTR, cardiac involvement or peripheral neuropathy is rarely observed in AA amyloidosis (6). Renal biopsy reveals moderate to severe glomerular involvement in all cases, most often including mesangial nodular amyloid deposits. Vascular involvement is seen in ∼95% of biopsies, in addition to interstitial and tubular basement membrane involvement. IHC-based staining for amyloid A can be used for diagnostic confirmation (6).

Treatment of AA amyloidosis depends on adequate control of the underlying inflammatory disorder, and measurement of SAA concentration can be used to assess response to treatment (37).

### Leukocyte chemotactic factor 2 amyloidosis (ALECT2)

Amyloidosis caused by leukocyte chemotactic factor 2 (ALECT2) was recently described but is already the third cause of renal amyloidosis in the United States (17,38). It predominantly affects Hispanic, Egyptian, Indian, and Pakistani individuals and manifests as chronic renal dysfunction of unclear etiology. No pathogenic variants have been described to date (39,40). Indeed, in a case series of ALECT2, none of 72 patients had a family history of amyloidosis and no pathogenic variants were detected in *LECT2* in the 16 patients analyzed at the molecular level (41). ALECT2 affects mainly the cortical interstitium in a diffuse pattern, leading to interstitial inflammation, while medullary involvement is minimal or absent. This lesion is sometimes confused with interstitial fibrosis. Glomerular involvement tends to be mild, thus proteinuria is variable and rarely reaches nephrotic levels. A recent study reported a median estimated time from diagnosis to ESKD of 8.2 years (39). Analysis by IHC can aid in diagnosis, but alone may not be sufficient to make the diagnosis of LECT2 amyloidosis in cases with weakly positive staining (17,40).

Currently, no specific treatment for ALECT2 exists. Kidney transplantation (KT) appears to be a reasonable therapeutic option for patients with ESKD, but the disease may recur in the allograft (41).

### Hereditary amyloidosis

Hereditary amyloidoses account for ∼10% of systemic amyloidosis cases and result from pathogenic variants in several genes whose corresponding protein products acquire amyloidogenic features (7,8). While AL is the most common subtype of amyloidosis, misdiagnosis is frequent: among 350 cases with preemptive diagnosis of AL amyloidosis evaluated in the United Kingdom, genetic and specific IHC analyses revealed that 34 (9.7%) had hereditary amyloidosis, eight of whom (24%) had evidence of monoclonal gammopathy (42–44). It is important to highlight that these diseases display autosomal dominant inheritance with variable penetrance and should be included in the differential diagnosis of amyloidosis even in the absence of family history (8).

### Transthyretin amyloidosis

There are two forms of transthyretin amyloidosis (ATTR), which result from amyloidogenic transthyretin (TTR) proteins: the wild-type ATTR (ATTRwt) amyloidosis, formerly known as senile systemic amyloidosis, with a dominant cardiac phenotype and associated with aging; and the hereditary ATTR amyloidosis, also known as ATTRv, caused by pathogenic variants in the *TTR* gene. Although ATTRv is the most common form of hereditary amyloidosis, it is still a rare disorder.

ATTRv was initially identified in Portuguese families. It is characterized by peripheral neuropathy and autonomic dysfunction, having previously been known as familial amyloidotic polyneuropathy. More than 120 pathogenic variants in the *TTR* gene have been described to date, among which Val30Met is the most common (45). Clinical manifestations begin around the third or fourth decades of life, including mainly sensory-motor polyneuropathy of extremities, autonomic neuropathy, with gastrointestinal symptoms (diarrhea and/or constipation), neurogenic bladder and erectile dysfunction, and non-specific symptoms, such as unintentional weight loss, and early satiety (46). It is an inexorably progressive and irreversible condition. Cardiac involvement may remain latent or undiagnosed, often leading to syncope, heart failure with preserved ejection fraction, and atrioventricular block (45,46).

Renal involvement in ATTRv is observed in only 15 to 20% of cases (47). The amyloid protein can be identified by IHC (48). Renal dysfunction or proteinuria does not seem to correlate with age, disease duration, or severity of neuropathic involvement. Notably, amyloid deposition has been described in glomeruli, vessels, and interstitium even in patients without clinical evidence of renal involvement. Microalbuminuria represents the first stage in renal involvement, and up to half of the patients with microalbuminuria may progress to renal dysfunction within two years (49). Among 403 patients with ATTRv, up to a third presented with proteinuria and 10% progressed to ESKD within 5 to 10 years of onset of albuminuria (50).

ATTRv should be suspected in patients with red-flag signs/symptoms such as peripheral polyneuropathy with no defined etiology, restrictive cardiomyopathy or atrioventricular block, and renal dysfunction/proteinuria with no apparent cause. *TTR* gene sequencing is the gold standard for diagnosis (46).

ATTR and AL often affect the heart, and the distinction between these two diagnoses may be challenging. Scintigraphy using technetium-^99m^ (e.g., ^99m^Tc-pyrophosphate)-labeled bone tracers is helpful to detect cardiac amyloid and may help to differentiate ATTR from AL. ATTR cardiac amyloid typically shows avid myocardial uptake of the ^99m^Tc-labeled tracer whereas the uptake is classically low by AL cardiac amyloid (51).

Recently, several disease-modifying agents focusing on the amyloidogenic process have been demonstrated, including the use of RNA-targeted therapies that interfere with hepatic TTR synthesis and agents that reduce formation of TTR amyloid (e.g., patisiran and diflunisal), preventing release of amyloidogenic monomers (46). Liver transplantation has been considered a therapeutic option to cure ATTRv (52).

### Fibrinogen A α-chain amyloidosis

Fibrinogen A α-chain amyloidosis is the most common hereditary renal amyloidosis in Europe. To date, 15 amyloidogenic *FGA* (fibrinogen alpha chain) gene variants have been identified. AFib amyloidosis was originally described in 1993 in a Peruvian kindred as a result of the Arg573Leu *FGA* variant, however the most commonly reported amyloidogenic variant is Glu545Val (E545V) (53). In Brazil, the first patient with AFib amyloidosis was reported in 2013, harboring the Glu545Val pathogenic variant (54).

Patients are affected after the third decade of life (mean age of 55 years) and present with hypertension, proteinuria, or nephrotic syndrome with rapid progression to ESKD approximately 5 years after diagnosis. Despite its autosomal dominant inheritance, a family history of amyloidosis and/or CKD has been found in only 64% of cases (19). Renal biopsy shows massive deposition of amyloid material in glomeruli, causing distortion and collapse of capillary loops. In contrast to this intense glomerular involvement, the interstitium and vessels display little or no deposition. Renal medulla is not affected. IF is negative for light chains, while IF or IHC staining for fibrinogen can be used for diagnostic confirmation.

There is no specific treatment for the disorder. The Glu545Val variant is usually associated with late-onset disease, slow progression to ESKD, low penetrance, and a good outcome after KT. *FGA* frameshift variants, on the other hand, are associated with early onset disease, fast progression, and fast amyloid recurrence after KT (7,18,19,54). In a recent French case series, KT appeared to be a viable option for patients with the Glu545Val variant, whereas liver-kidney transplant may be a preferred option in patients with frameshift variants (18).

### Lysozyme amyloidosis

Lysozyme amyloidosis is extremely rare, usually presenting with renal, hepatic and/or intestinal involvement and/or Sjögren's syndrome. Renal involvement includes progressive renal dysfunction and nephrotic proteinuria (55). Renal biopsy shows extensive amyloid deposition in mesangium, capillary loops, and blood vessels. Medullary amyloid deposition can also be seen along the basement membrane of the collecting ducts and vasa recta. IF and IHC can be used to confirm the diagnosis (56). Renal disease progression varies between patients. In a retrospective evaluation, the median time from discovery of renal dysfunction to ESKD was 11 years (57). Kidney transplant may be useful for patients with end-stage kidney failure (57).

### Apolipoprotein amyloidosis

Mutated apolipoproteins AI, AII, CII, and CIII, as well as wild-type apolipoprotein AIV, can serve as precursors for different types of amyloid molecules. AApoAI and AApoAIV amyloidoses manifest around the sixth decade of life with slow and progressive loss of renal function, associated with mild or absent proteinuria (3,7). Notably, pathogenic variants in the *APOA4* (apolipoprotein A4) gene have not been detected. Renal biopsy shows marked amyloid deposition in renal medulla along with tubulointerstitial nephritis, while glomeruli are almost always spared (58).

In contrast, patients with ApoAII and ApoCII renal amyloidoses present with renal dysfunction and proteinuria, which may reach a nephrotic range (59,60). These amyloidoses also affect the liver, skin, heart, and adrenals. Renal biopsy in AApoAII shows extensive deposition of amyloid material in mesangium and capillary loops, while in AApoCII there is predominantly glomerular involvement with asymmetric mesangial expansion with mesangial nodules. The interstitium is typically spared, but larger vessels may be involved (61).

AApoCIII amyloidosis is a recently described form of renal amyloidosis (62). Its manifestations include tubulointerstitial kidney disease, Sjögren's syndrome at the age of 20 years, and progressive renal insufficiency. Amyloid deposition was identified in the renal cortex, glomeruli with extensive mesangial distribution, peritubular basement membranes, and renal interstitium. Deposition of amyloid fibrils affected mostly the vascular compartment with abundant amyloid deposits in the wall of renal arterioles leading to lumen obliteration (62,63). There is no specific treatment for the disease. Kidney transplantation is a therapeutic option for patients with ESKD.

### Gelsolin amyloidosis

Gelsolin is an actin-modulating protein present in cytosolic and secretory forms in most cells, with roles in multiple biological processes. Mutant forms can rarely cause amyloidosis (64). The most common clinical signs of AGel amyloidosis are progressive corneal lattice amyloidosis, cranial and peripheral neuropathy, and cutis laxa lesions, typically manifested in the fourth or fifth decades (65). Proteinuria and renal failure were detected in 13 and 5% of the patients, respectively (65). While renal involvement usually manifests as mild and intermittent proteinuria in heterozygotes for pathogenic variants in the *GSN* (Gelsolin) gene, homozygous patients for such variants present significant proteinuria and may develop nephrotic syndrome as early as in their early twenties (66).

## Histological diagnosis

The finding of positive birefringence (apple green) in tissue samples stained with Congo red under polarized light is the gold standard for diagnosis of amyloidosis, regardless of the affected organ but not specific for the amyloid subtype. Histological evaluation of the affected organ is the most sensitive method for diagnosis, although the assessment of other sites such as abdominal fat pad, rectal mucosa, or salivary glands can be pursued. In non-AL amyloidoses, however, this last approach is associated with low sensitivity.

**Figure 2:**
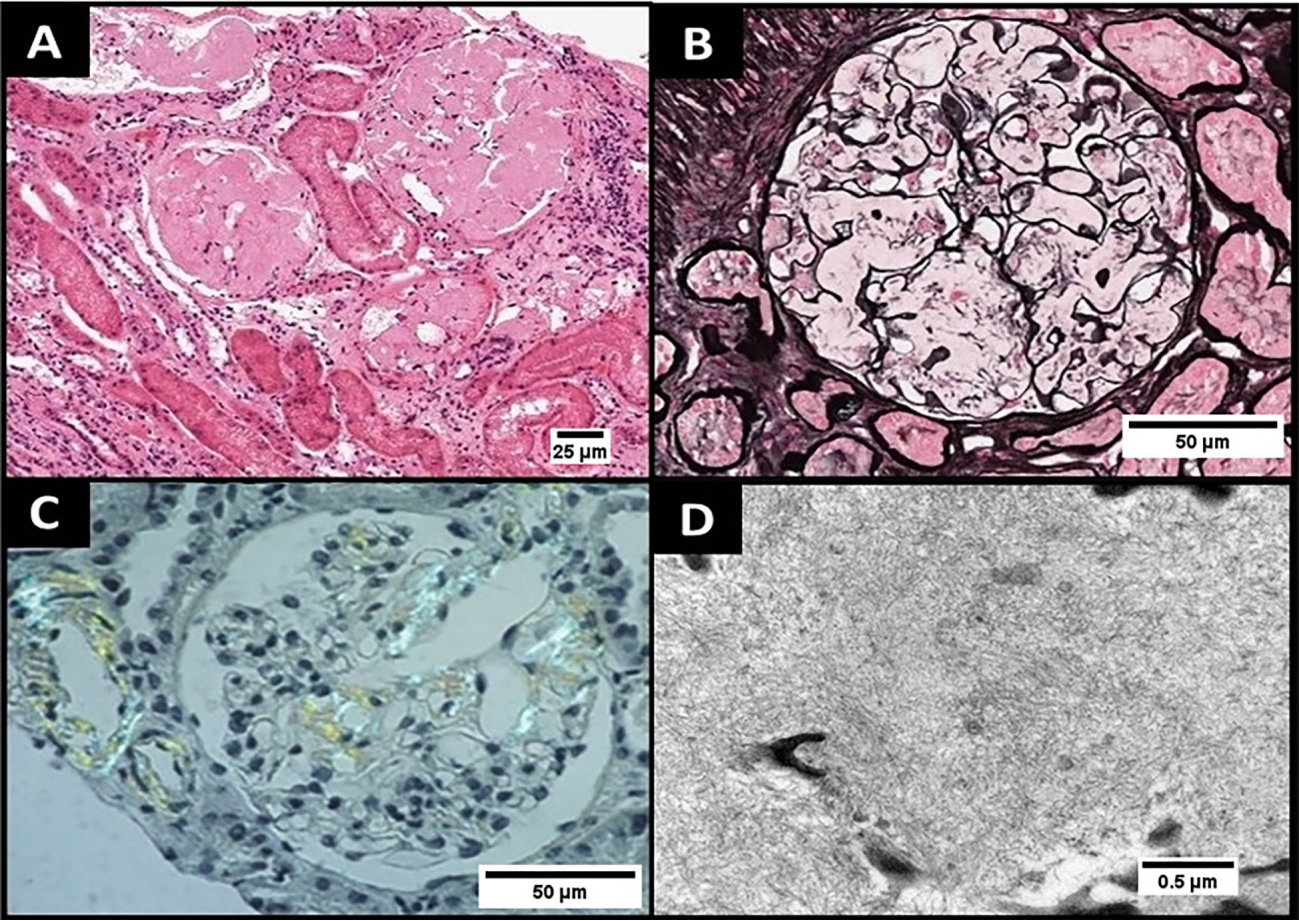
Kidney biopsy from a patient with Ig light chain (AL) amyloidosis. **A**, Glomerular architecture reveals amorphous, eosinophilic deposits located in the mesangium and subendothelial space (hematoxylin and eosin, original magnification; 200×, scale bar 25 µm). **B**, Glomerular deposits exhibit little or no affinity for silver (silver methenamine; 400×, scale bar 50 µm). **C**, Staining by Congo red (original magnification; 400×, scale bar 50 µm), showing positive birefringence under polarized light. **D**, Ultrastructural studies show deposition of thin fibrils, usually between 8-12 nm, with random distribution (60,000×, scale bar 0.5 µm). Images are a courtesy from Lívia Barreira Cavalcante, MD (Divisão de Patologia, Universidade de São Paulo, São Paulo).

Renal biopsy is often used for diagnosis and identification of amyloid precursors. Amyloid deposition occurs mainly in the glomeruli. On light microscopy, the amyloid material deposited in the glomerulus appears as amorphous material in the mesangium and capillary loops. Mesangial deposition can be intense and produce an amorphous hyaline appearance with a nodular configuration on hematoxylin-eosin staining. This takes on a salmon color when stained with Congo red. While the nodular aspect can raise the differential diagnoses of diabetic kidney disease and light chain deposition disease, the amyloid protein predominates in tissues affected by amyloidosis, with reduced collagen deposition in the extracellular matrix evidenced by weak staining with periodic acid-Schiff (PAS) (Figure 2) (17,27). Tubulointerstitial deposits of amyloid, in contrast, produce tubular atrophy and interstitial fibrosis.

On electron microscopy (EM), the amyloid material appears as randomly deposited unbranched fibrils without specific orientation in the mesangium, vessels, and/or interstitium. The fibril size helps differentiate between amyloidosis and other renal deposit diseases, such as fibrillary glomerulonephritis and immunotactoid glomerulonephritis. In these diseases, fibrils have a diameter of 15 to 20 nm and 30 to 90 nm, respectively. Furthermore, the appearance of amyloid fibrils on EM is characteristic, establishing the diagnosis even when Congo red staining is negative, which may occur in up to 5-13% of cases (7,21,27).

## Identifying the amyloid precursor protein and/or the primary disorder

Accurate identification of the specific amyloid protein of systemic amyloidosis is necessary to assess prognosis, perform genetic counseling, and delineate the appropriate treatment. Exclusion of hereditary amyloidosis is often very useful in helping solidify the diagnosis of AL amyloidosis. Currently, immunodetection methods and laser microdissection/mass spectrometry are used alone or together to determine the amyloid fibril protein in every case of amyloidosis.

### Immunodetection methods

Typing the amyloid precursor is traditionally performed by immunodetection methods using specific antibodies, such as IF and/or IHC. IF is commonly used to document deposition of immunoglobulin proteins in the kidney and is typically performed on frozen sections of kidney biopsies. Fluorochrome-labeled monoclonal antibodies directed against immunoglobulin, complement, and fibrinogen are incubated with tissue-sample slides and viewed under a fluorescence microscope (67). IHC studies use the same principle. Use of specific antibodies can aid in typing the amyloid in renal AL and non-AL amyloidosis. The use of other antibodies such as anti-serum amyloid A, anti-transthyretin, or anti-LECT2 are also available by IHC (Table 3) and can be performed to confirm the diagnosis of these different subtypes of amyloid proteins (21,67).

**Table 3:** Diagnosis of renal amyloidosis: pathology assessment and tests to identify amyloid precursors.

Renal amyloidosis	Precursor protein	Renal pathology			LMD/MS	Genetic testing
		Light microscopy	IF	IHC		
AL/AH/AHL	Immunoglobulin light and/or heavy chain	Deposition in glomeruli, vessels, and interstitium	Sensitivity 85% and specificity 92% (Ref. 71) for Ig	AvailableDefinitive results are obtained in <60% of cases (Ref. 80)	>97% accuracy (Ref. 17)	Not available
AA	Serum amyloid A	Glomeruli always affected. Vascular and interstitium involvement are common	Negative	Available	Equivocal cases	Not available
AFib	Fibrinogen A α-chain	Massive glomerular deposition. Medulla and vessels not involved	Positive for fibrinogen	AvailableNot definitive in ∼10% (Ref. 81)	Inconclusive cases	Available
ALECT2	Leukocyte chemotactic factor 2	Deposition in glomeruli, vessels, and interstitium	Negative	AvailableHigh false-positive rate (Ref. 40)	LMD/MS to avoid inaccurate diagnosis(Ref. 40)	Not applicable
ATTR	Transthyretin	Deposition in glomeruli, vessels, and interstitium	Negative	Available	Inconclusive cases	Available
ALys	Lysozyme	Deposition in glomeruli, vessels, and interstitium	Negative	Available	Inconclusive cases	Available
AApoAI	Apolipoprotein AI	Deposition in inner medulla. Interstitial nephritis	Negative	Available	Inconclusive cases	Available
AApoAII	Apolipoprotein AII	Deposition in glomeruli and vessels	Negative	Available	Inconclusive cases	Available
AApoAIV	Apolipoprotein AIV	Deposition restricted to renal medulla. Cortex is spared	Negative	Available	Inconclusive cases	Not applicable
AApoCII	Apolipoprotein CII	Predominantly glomerular and medullary involvement. Minimal vessels/interstitial involvement	Negative	Not available	Inconclusive cases	Available
AApoCIII	Apolipoprotein CIII	Deposition in glomeruli, vessels, and interstitium. Interstitial nephritis	Negative	Available	Inconclusive cases	Available
AGel	Gelsolin	Restricted to glomeruli, spares vessels and interstitium	Negative	Available	Inconclusive cases	Available

IF: immunofluorescence; IHC: immunohistochemistry; LMD/MS: laser microdissection/mass spectrometry; Ig: immunoglobulin. Ref 17: doi: 10.2215/CJN.10491012; Ref 40: doi: 10.1016/j.humpath.2014.02.020; Ref 71: doi: 10.1016/j.kint.2019.05.027; Ref. 80: doi: 10.1111/bjh.13156; Ref 81: doi: 10.1681/ASN.2008060614.

AL amyloid includes the variable domain of immunoglobulin κ or λ light chains. Because of sequence variability of this domain and alterations in the structure of the light chains in the amyloid, antibodies raised against intact light chains may not react to an amyloid fibril. Conformational or fragmentation changes can mask or eliminate specific epitopes and obscure the diagnosis (27). Additionally, since antibodies are typically developed against the constant region, limited or nonreactivity would be expected. In a small percentage of cases with AL-amyloidosis (about 10-15%), commercially available antibodies do not detect the mutated light chains (27,68).

Immunoelectron microscopy (IEM), in turn, combines IHC and EM to identify amyloid with greater sensitivity and specificity (69). A study analyzed 423 cases of amyloidosis with IEM and appropriately identified the specific type in more than 99% of cases (70). Challenging and inconclusive cases can be accurately typed using IEM, but the lack of expertise often prevents a wider use (69).

IF generally has better accuracy than IHC and is more frequently used to diagnose AL amyloidosis (71). Amyloid A, transthyretin, fibrinogen, and LECT2 can be detected by IHC. In ∼20-25% cases, however, IHC fails to prove the amyloid subtype, since the techniques and the diagnostic sensitivity and specificity significantly vary among centers (72). The absence of light chain reactivity in IF or IHC does not exclude the diagnosis of AL amyloidosis; moreover, IF labeling for kappa or lambda chains in renal tissue may be negative in 14 to 35% of AL cases.

Identification of monoclonal kappa or lambda light chain in AL is sufficient for diagnosis in the setting of evident plasma cell dyscrasias. With the support of IF and IHC, renal biopsy analysis can establish the diagnosis of AL and AA amyloidosis in most cases, but these methods have limited specificity and sensitivity (Table 3). In the absence of accurate identification of the amyloid precursor, the diagnosis remains undetermined requiring further investigation.

### Laser microdissection/mass spectrometry

Laser microdissection/mass spectrometry (LMD/MS) is a relatively new technique used to diagnose and type amyloidosis. The protein profile of Congo red-positive dissected areas of a kidney biopsy is typically analyzed. Laser microdissection (LMD) is initially used to capture pure amyloid plaques (in the glomeruli, vessels, and/or interstitium) from routine formalin-fixed and paraffin-embedded tissues, enhancing the specific signal while significantly reducing background tissue and serum contamination (9).

Tissue samples are processed to extract the protein component. The proteins are then cut into peptides using trypsin, an enzyme that cleaves them at lysine and arginine residues, yielding a peptide complex thereafter separated by liquid chromatography (LC). After separation by LC, the solution is exposed to high voltage and generates ionized peptides that are sprayed into the mass spectrometer. In tandem mass spectrometry (MS/MS) analysis, the first MS measures the mass-to-charge (m/z) ratio of the parent peptide. Peptides selected by predetermined criteria are then directed to a collision cell for fragmentation upon impact with an inert gas such as helium, a process called collision-induced dissociation (CID). The fragments formed through CID are measured in the second MS. Tryptic peptides present in the human proteome have unique CID patterns, which allow the prediction of their amino acid sequences. A number of complex computational algorithms have been developed to predict such sequences. Algorithms are used to compare the fragmentation pattern of each peptide to the theoretical fragmentation pattern of all human tryptic peptides predicted by the reference human genome. Based on this comparison, they allow the assignment of a probability score for individual peptides as well as the assignment of an identification probability score for a given protein, based on the fact that multiple peptides are derived from an initial protein (9,20,73). As an alternative method to Congo red staining, amyloidosis can be diagnosed on the basis of proteomics using LMD to identify the presence of serum amyloid-P component and apolipoprotein E. MS-based proteomics can be applied to identify any of the amyloid subtypes should they be acquired or hereditary (20).

This approach has been employed for diagnoses in cases initially classified as indeterminate. A recent study compared the sensitivity and specificity of IF to LDM/MS in 170 cases of renal biopsies from patients with amyloidosis (71). One hundred and four cases were identified as Ig-amyloidosis and 66 as non-Ig amyloidosis. Compared to LDM/MS, the sensitivity and specificity of IF were 84.6%and 92.4%, respectively. IF failed to identify the amyloid protein in 12.3% of the cases with renal amyloidosis, revealing lower sensitivity and specificity than LMD/MS (71).

Some advantages of LMD/MS over conventional methods of amyloid typing are: a) LMD/MS is a single test that can identify the amyloid protein in question; b) one can avoid multiple IHC tests for amyloid typing; c) LMD is performed directly on the involved tissue (Congo red-positive areas are isolated), which increases precision and reduces contamination by other potentially amyloidogenic proteins; d) LMD/MS is performed on paraffin-embedded material and does not require frozen specimens, which allows re-evaluation of stored tissue samples; e) familial and hereditary amyloidosis are difficult to diagnose on routine IF and IHC studies, while LMD/MS is remarkably useful and sensitive in diagnosing and typing cases for which elaborate, time-consuming genetic analyses are required; and f) monoclonal gammopathy may be present in patients with non-AL amyloid, whereas LMD/MS is extremely effective at confirming hereditary or other types of amyloid in such cases, and at ruling out AL amyloid in the setting of a monoclonal gammopathy (17,20,71).

LMD/MS is therefore the current diagnostic tool with the highest diagnostic accuracy, establishing itself as the gold standard method to determine the amyloid precursor. Its use will enhance our ability to type amyloidosis accurately in clinical biopsy specimens. Currently, however, this method has limited availability and a high cost, thus is most often reserved for negative IF/IHC or inconclusive cases.

## Genetic testing

Familial forms of amyloid-related diseases are often associated with specific variants in the gene encoding the peptide/protein that aggregates into amyloid deposits. A genetic cause for amyloidosis should be considered for every patient, as many cases have no family history due to incomplete penetrance, unrecognized onset, or death from other causes in previous generations (42). Genetic testing of all patients is inappropriate due to cost and time but should be considered in cases where hereditary amyloidosis is suspected or cannot be ruled out based on phenotype (74).

Testing for most hereditary forms of amyloidosis (ATTRv, AFib, ApoAI, and ALys) is available (Table 3). After identifying the involved protein through IF, IHC, and/or LMD/MS, genetic tests can be performed to identify causative variants and screen at-risk family members. Whole exome sequencing is now used in the diagnosis of Mendelian disorders and can be utilized in cases of amyloidosis that were not diagnosed by the previous tests (75).

## Diagnostic approach in renal amyloidosis

**Figure 3:**
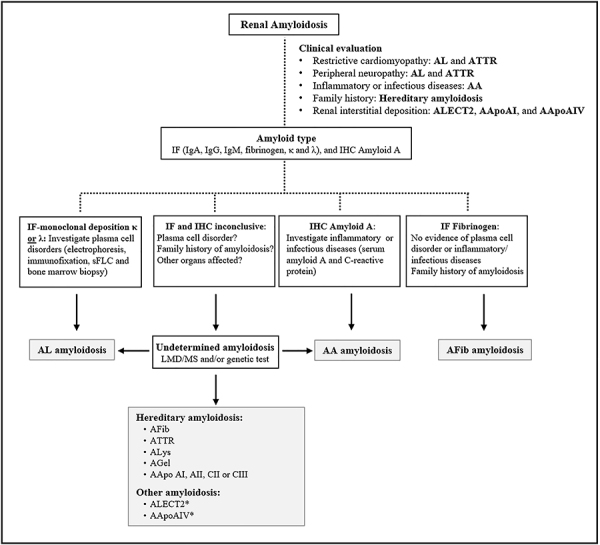
Flowchart of investigation and diagnosis of renal amyloidosis. IF: immunofluorescence; IHC: immunohistochemistry; LMD/MS: laser microdissection/mass spectrometry; Ig: immunoglobulin. *No pathogenic variants have been described to date.

The investigation of patients with suspected systemic amyloidosis requires careful clinical assessment, in addition to complementary tests to identify the affected organs/systems. The involvement of specific organs evidenced by particular signs, symptoms, and tests can determine the biopsy site and subsequent diagnostic investigation (Figure 3). A history of chronic inflammatory or infectious diseases should guide the etiological investigation towards serum amyloid A. Cases of renal amyloidosis with a history of poorly controlled, chronic inflammatory disease are strongly suggestive of AA amyloidosis. Findings of amyloid A deposition by IHC and absence of monoclonal Ig by IF are sufficient to establish the diagnosis. In up to 20% of cases, however, IF and IHC are inconclusive, requiring further assessment (72).

Macroglossia or periorbital purpura, although less common, strongly suggests AL amyloidosis. In patients with suspected amyloidosis and proteinuria, in whom histological assessment of more accessible sites (e.g., abdominal fat pad aspirate) is not possible or was non-diagnostic, a renal biopsy should be performed to confirm the diagnosis and, subsequently, identify the precursor protein through IF or IHC. It is not uncommon that patients with histological evidence of amyloidosis and monoclonal gammopathy be automatically classified as AL amyloidosis. However, in such cases, it is essential to identify the monoclonal light chain in the evaluated tissue to accurately establish the diagnosis. In addition, as the lack of light chain identification in the amyloid material does not exclude the diagnosis of AL amyloidosis, cases with suggestive electrophoresis, immunofixation, sFLC dosage, and/or bone marrow evaluation should be confirmed by LMD/MS (71).

Monoclonal gammopathy of undetermined significance (MGUS) is a premalignant condition, characterized by the presence of low titers of monoclonal protein in the serum or urine without any related organ damage. MGUS most often affects older individuals, with an estimated prevalence of 3.2, 5.3, and 7.5% in individuals aged ≥50, ≥70, and ≥85 years, respectively (76). The possibility of underdiagnosis of non-AL types of amyloidosis should be highlighted. Detection of monoclonal gammopathy *per se* does not meet diagnostic criteria for AL amyloidosis (Table 4); interestingly, a high prevalence of MGUS (10-49%) has been observed in patients with ATTR (77). This reality may lead to diagnostic confusion with AL amyloidosis and patient mismanagement, leading to inappropriate chemotherapy and/or hematopoietic cell transplantation (78).

**Table 4:** International Myeloma Working Group diagnostic criteria for systemic AL amyloidosis (Ref. 82; doi: 10.1200/JCO.2015.65.0044).

Diagnosis of systemic AL amyloidosis requires all of the following:
a. Presence of an amyloid-related systemic syndrome (e.g., renal, liver, heart, gastrointestinal tract, or peripheral nerve involvement)
b. Positive amyloid staining by Congo red in any tissue (e.g., fat aspirate, bone marrow, or organ biopsy)
c. Evidence that amyloid is light-chain-related established by direct examination of the amyloid using mass spectrometry-based proteomic analysis, or immunoelectron microscopy
d. Evidence of a monoclonal plasma cell proliferative disorder (serum or urine monoclonal protein, abnormal free light-chain ratio, or clonal plasma cells in the bone marrow)

Hereditary amyloidosis should be suspected regardless of a positive family history of nephropathies or systemic amyloidosis. Sensorimotor polyneuropathy and cardiac involvement associated with a positive family history should guide investigation for ATTRv amyloidosis (46). A diagnostic delay of up to four years has been observed in regions that are non-endemic for this disorder (79). Upon initial clinical suspicion, genetic testing directed at suspected genes should be performed promptly.

## Conclusion

Amyloidosis is a multisystemic disease that frequently affects the kidneys and manifests as nephrotic syndrome. Following clinical suspicion, diagnosis should be established by histological assessment using Congo red staining, which, under polarized light, yields positive birefringence. As a subsequent diagnostic step, the amyloid precursor protein should be identified.

In patients with renal amyloidosis, identifying a monoclonal protein by serum and urine protein electrophoresis and/or immunofixation is not sufficient to establish a diagnosis of AL amyloidosis. Moreover, the absence of light chains by IF and IHC does not exclude this diagnosis. Although AL is the most frequent subtype of amyloidosis, inconclusive diagnoses are frequent. In this setting, the diagnosis of other types of amyloidosis, especially hereditary forms, has been significantly expanded with the greater availability of genetic tests and LMD/MS. Inconclusive cases should be referred to reference centers with broader diagnostic methodologies in order to guide appropriate treatment and avoid potentially harmful therapies.
